# A case of esophageal carcinoma on esophageal varices treated by endoscopic submucosal dissection after endoscopic injection sclerotherapy

**DOI:** 10.1002/deo2.202

**Published:** 2022-12-30

**Authors:** Kenichiro Nakachi, Takao Maekita, Hisanobu Deguchi, Masatomo Kimura, Mikitaka Iguchi, Satoshi Yoshida, Masayuki Kitano

**Affiliations:** ^1^ Gastroenterological Medicine Kameda Medical Center Kamogawa Chiba Japan; ^2^ Department of Gastroenterology Wakayama Medical University Wakayama Japan; ^3^ Internal Medicine Nokami Kousei Sogo Hospital Wakayama Japan; ^4^ Department of Pathology Hashimoto Municipal Hospital Wakayama Japan; ^5^ Gastroenterological Medicine Hashimoto Municipal Hospital Wakayama Japan

**Keywords:** early esophageal squamous cell carcinoma, endoscopic injection sclerotherapy, endoscopic submucosal dissection, esophageal varix, esophagogastroduodenoscopy

## Abstract

Esophageal varices (EVs) are often treated using endoscopic injection sclerotherapy. Endoscopic submucosal dissection (ESD) has been used for early esophageal epithelial neoplasia worldwide. We report a case of early esophageal squamous cell carcinoma (ESCC) that occurred over EVs, in which the EVs were treated with endoscopic injection sclerotherapy before the early ESCC was treated with endoscopic submucosal dissection. Argon plasma coagulation was finally performed to prevent the recurrence of varices. No serious complications, such as severe bleeding or perforation, were observed. Histopathological examination revealed submucosal veins occluded with an organized thrombus for which endoscopic injection sclerotherapy with an intravariceal injection of sclerosant had been performed, but no fibrosis was observed outside the blood vessels. This explains that the injected sclerosant into EVs did not cause any tissue reaction like fibrosis in the submucosa surrounding the vein, which may have made endoscopic submucosal dissection safer and easier. Varices have not recurred, and ESCC has also not recurred for 5 years. We demonstrated a successful treatment of ESCC on EVs and no submucosal fibrosis other than inside the occluded vessels and verified it histologically.

## INTRODUCTION

Alcohol is a significant risk factor for the development of esophageal squamous cell carcinoma (ESCC) and liver cirrhosis. Individuals with liver cirrhosis may subsequently develop esophageal varices (EVs). Endoscopic submucosal dissection (ESD) is an effective treatment for early (ESCC), but bleeding is a serious complication of ESD. No consensus has yet been reached regarding the most suitable method of treating EVs concomitant with early ESCC.

## CASE REPORT

**FIGURE 1:**
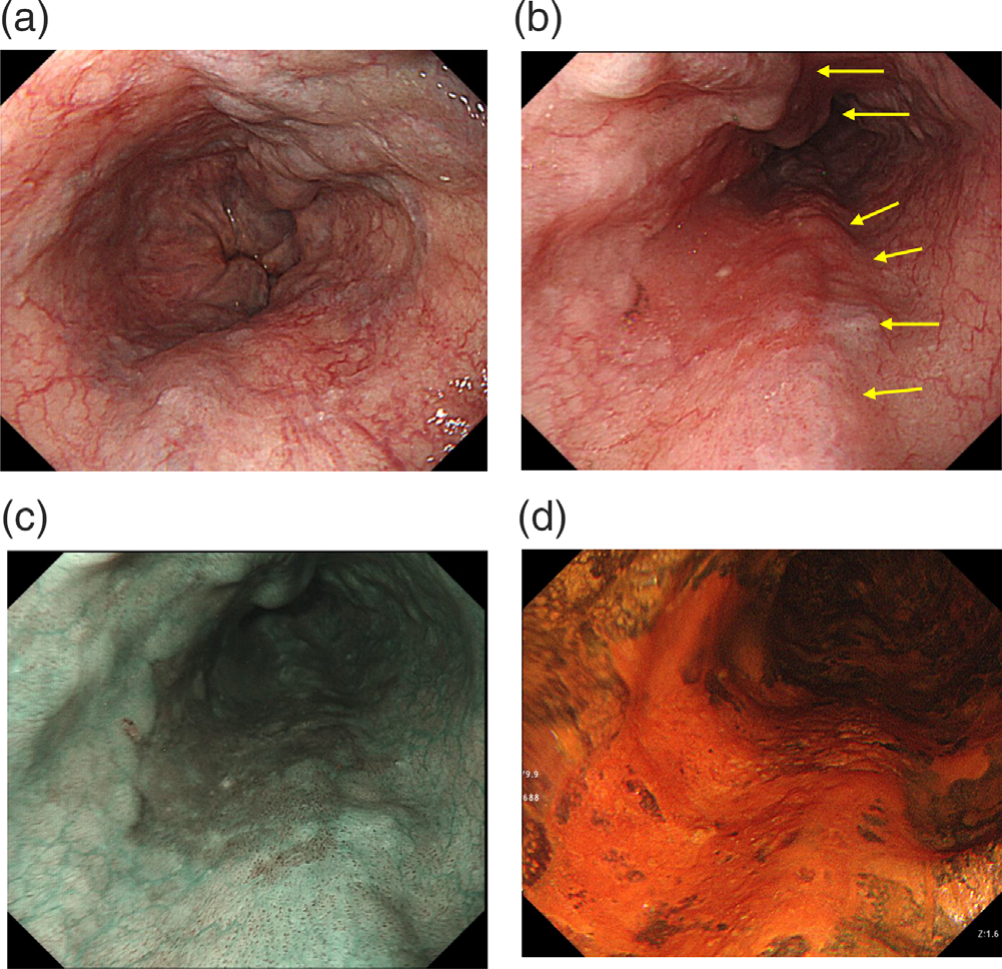
Results of esophagogastroduodenoscopy: (a) beaded varicose veins on the lower thoracic esophagus; (b) superficial carcinoma, type 0–IIb, with a reddened lesion, 3 cm in length and with varices (yellow arrows) beneath and near the lesion on the middle thoracic esophagus; (c) using narrowband imaging (NBI), the line demarcating the lesion is clear, and a brownish area is identified; (d) unstained lesion with Lugol 1.0% chromoscopy

A 66‐year‐old man had been diagnosed with alcoholic cirrhosis and EVs. Cirrhosis was classified Child‐Pugh‐B7, and three beaded varices had developed. The patient stopped drinking alcohol and started liver treatment. After 6 months, cirrhosis improved to Child‐Pugh‐A6. Esophagogastroduodenoscopy detected a reddish, 0–IIb, 3‐cm lesion on EVs in the middle intrathoracic esophagus. This lesion was recognized as a well‐demarcated, brownish area on narrowband imaging. Chromoendoscopy using Lugol's solution showed the lesion as an unstained orange area (Figure 1). Biopsy revealed squamous cell carcinoma, whereas computed tomography of the chest and abdomen revealed no metastases.

We considered that an endoscopic resection of the lesion should be left until after the treatment of the EVs to prevent bleeding during resection. We chose to perform endoscopic injection sclerotherapy (EIS) first.

**FIGURE 2:**
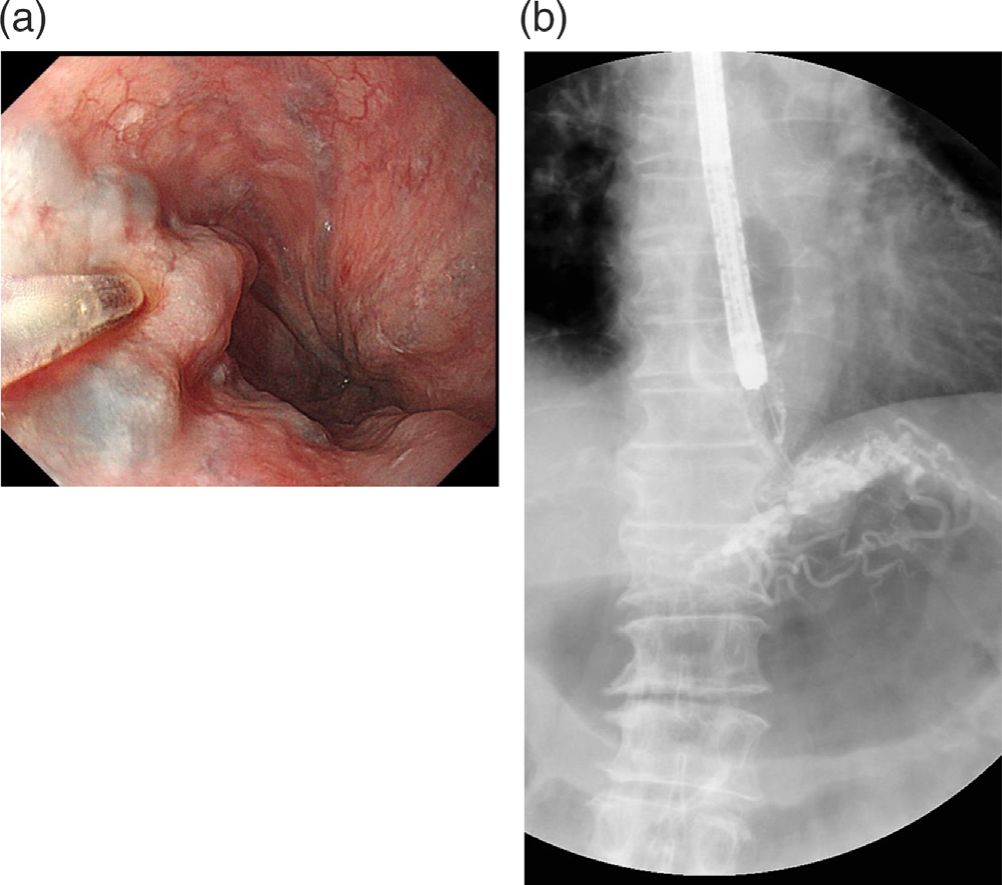
(a) Endoscopic injection sclerotherapy performed under a freehand technique using a flexible needle injector inserted through the biopsy channel of the endoscope. Injection of 5% monoethanolamine oleate caused an acute inflammatory reaction in the intimal endothelium of the vein. (b) Fluoroscopic examination of the injection with 21 ml of 5% monoethanolamine oleate

A GIF‐H260 electronic endoscope system (Olympus, Tokyo, Japan) was used for EIS. Sclerosant (10% monoethanolamine oleate; Fuji Chemical Industry, Toyama, Japan) was mixed with an equivalent volume of iopamidol (Hikari Pharmaceutical Co., Tokyo, Japan). The initial procedure for EIS was performed with an intravariceal injection of sclerosant under X‐ray fluoroscopy from the lower intrathoracic esophagus and esophagogastric junction. EIS was performed using a freehand technique with a flexible needle injector (Top Corporation, Tokyo, Japan) inserted through the instrument channel of the endoscope. The sclerosant (21 ml of 5% monoethanolamine oleate) was injected into the varices at a few centimeters closer to the anus than the tumor and stopped injecting when sclerosant flowed into the left gastric vein (Figure 2). When the needle was removed, pressure hemostasis was performed by the balloon. No EVL was performed. After 1 week, a second EIS was performed. However, the sclerosant (5% monoethanolamine oleate) could not be injected into varices. From this, we concluded that sufficient sclerosant had been injected to occlude the varices. A period of 2 weeks after the first EIS, 1% polidocanol (Kaigen Pharma Co., Osaka, Japan) was injected into five sites of the paravariceal submucosa on the anal side of the ESCC as intensive therapy, with 2 ml per injection.

**FIGURE 3:**
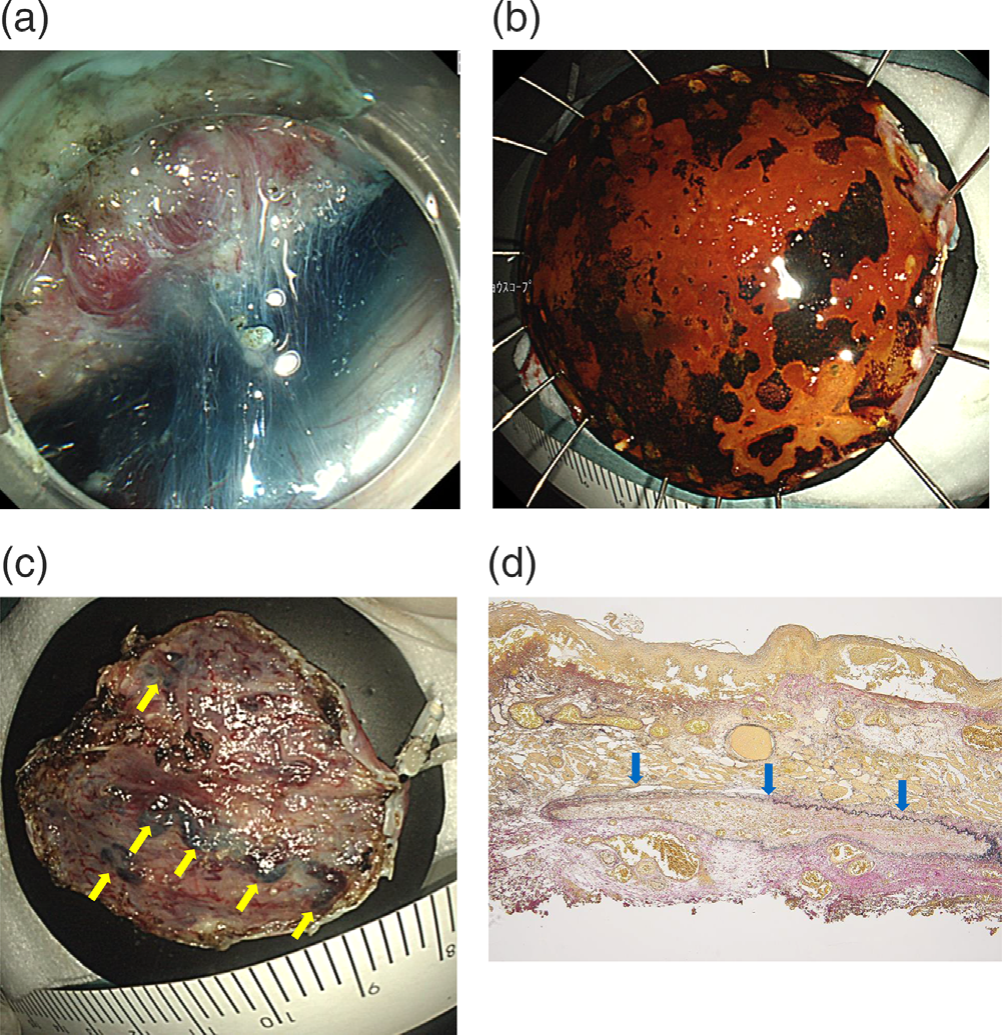
(a) During submucosal dissection immediately after mucosal incision, dilated veins and no fibrosis are recognized in the submucosa. (b) En bloc resection. (c) Varices are apparent in the dissected side of the specimen. Embolization of varices is recognized as bronze varices (yellow arrows). (d) A submucosal vein with intravascular organized thrombus. Adventitious elastic fibers (black colored) are evident surrounding the thrombosed vein (*blue arrows*). The intravascular thrombus has been organized with granulation tissue occluding the vein. No granulation tissue and fibrosis were found around the thrombosed vein (Elastica van Gieson stain).

A period of 4 weeks after first EIS, ESD was performed using a dual knife (Olympus) with the GIF‐HQ290 electronic endoscope system (Olympus) and an ICC200 electrosurgical generator system (ERBE Elektromedizin, Tübingen, Germany). No severe bleeding occurred during ESD. En bloc resection of this lesion was accomplished without serious complication (Figure 3). Histopathological examination revealed squamous cell carcinoma invading the lamina propria mucosae without any invasion of the lymphatic vessels or veins. Both horizontal and vertical margins were negative. Some submucosal veins were occluded with an organized thrombus. No extravascular granulation tissue and fibrosis were found.

**FIGURE 4:**
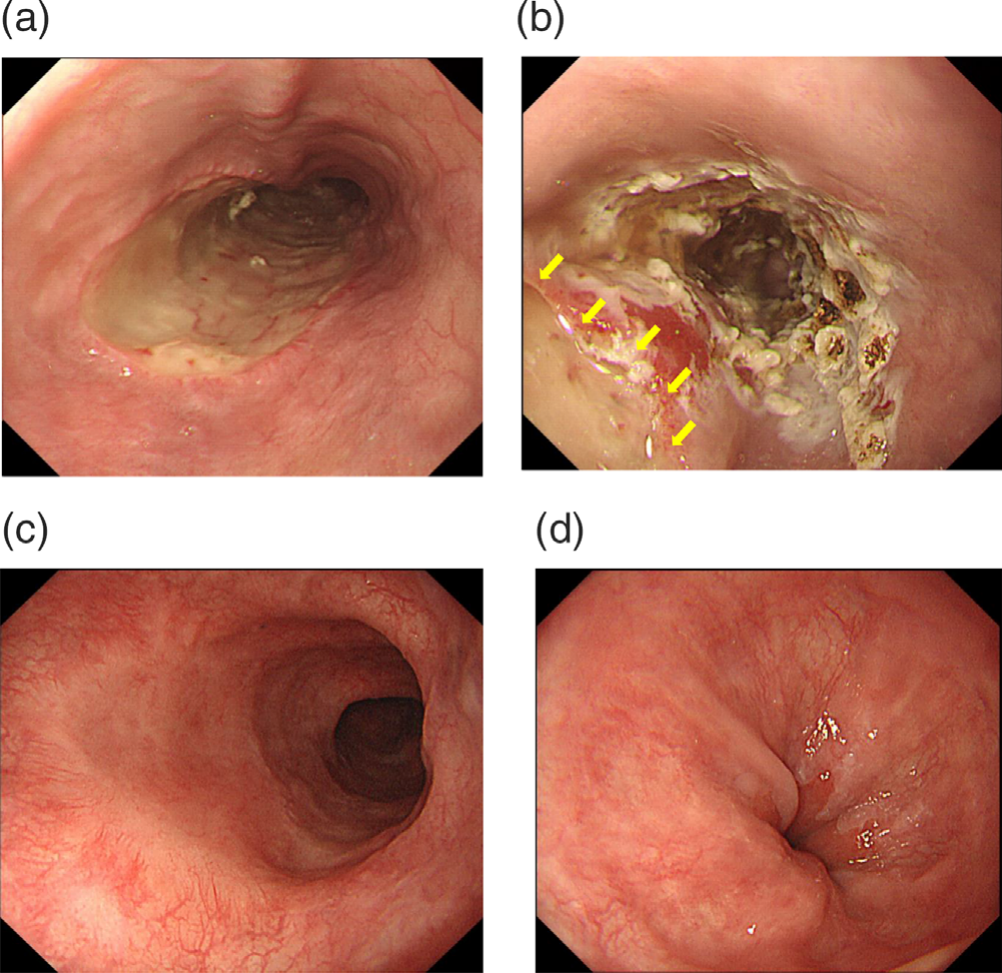
(a) The esophagus 2 weeks after endoscopic submucosal dissection (ESD). A white mosslike region is seen on the mucosal defect. (b) A period of 2 weeks after performing ESD, argon plasma coagulation is performed in the esophagus. Tissue coagulation is accomplished without coagulating the post‐ESD ulcer (*yellow arrow*). (c) Middle thoracic esophagus 5 years after ESD. The mucosal defect has healed, and no local recurrence has been identified. (d) The esophagogastric junction after 5 years. Varices have disappeared.

A period of 6 weeks after first EIS and 2 weeks after ESD, argon plasma coagulation (APC) was performed using the ICC200 electrosurgical generator, an APC300 automatically regulated argon source (ERBE Elektromedizin), and flexible APC probes (ERBE Elektromedizin) from the GIF‐H260 electronic endoscope system. The settings of the APC equipment were as follows: argon gas flow rate, 1.2 L/min; high‐frequency output, 50 W. Tissue coagulation was performed in the distal esophagus from 5 cm on the oral side of the esophagogastric junction to 1 cm on the anal side of the junction. Follow‐up esophagogastroduodenoscopy was performed every 6 months. As of 5 years after EIS and ESD, no recurrence of EVs or carcinoma has been seen (Figure 4).

## DISCUSSION

In the treatment of EVs, endoscopic variceal ligation (EVL) is usually the first choice in Western countries, whereas EIS remains the mainstay of varix eradication in Japan. EVL does not require outstanding levels of technical skill and has few complications. However, varices recur relatively frequently after EVL.^1^, ^2^ In contrast, EIS has a lower recurrence rate for EVs than EVL and improves prognosis, while requiring outstanding technical proficiency. In addition, EIS is contraindicated in cases with severe jaundice, severe hypoalbuminemia, severe thrombocytopenia, disseminated intravascular coagulation, massive ascites, severe encephalopathy, or severe renal dysfunction.^2^

The patient in the present case was able to completely stop drinking after being diagnosed with alcoholic cirrhosis. As a result, liver function improved and ascites resolved. Some cases of ESD after EVL have been reported that significant fibrosis was observed on the lesion around the band.^3^, ^4^ EVL sometimes retracted the wider mucosa than expected, and EVL scar and the tumor would be close or overlap. Accordingly, ESD would be difficult. We therefore performed EIS to treat the EVs, to avoid the difficulty of resecting lesions due to fibrosis and the risk of major bleeding if ESD resection had been performed first. While ESD after EIS, significant changes were not observed. Hyaluronic acid solutions as an injection solution were used to achieve sustained mucosal elevation, and the depth of dissection was far below varices. Consequently, appearance during ESD was not affected by thrombosed varicose veins. We could perform ESD normally.

Histopathological examination revealed submucosal veins occluded with an organized thrombus for which EIS with intravariceal injection of sclerosant had been performed, but no fibrosis was observed outside the blood vessels. This explains that the injected sclerosant did not cause any tissue reaction like fibrosis in the submucosa surrounding the vein, which may have made ESD safer and easier. Mochimaru reported that ESD after EVL and EIS with extravariceal injection caused severe bleeding and EIS with an intravariceal injection of sclerosant before ESD would be less likely to cause fibrosis than EVL.^4^ EIS with intravariceal injection without EVL requires advanced techniques, although it would be effective for subsequent ESD.

If the ESCC locates in the upper thoracic esophagus and EO is injected from the oral side, EO would tend to flow into the blood drainage routes and would not flow sufficiently into the blood supply routes. If the ESCC locates in lower esophagus or esophagogastric junction on EVs, EVL would be difficult and EIS injections on the anal side could be more effective.^5^

Generally, APC is performed 1–4 weeks after EIS.^6^ APC is usually a supplemental treatment for preventing recurrence of EVs. However, we prioritized ESD and performed APC 6 weeks after EIS.

For the management of ESCC, en bloc curative resection is considered ideal for both endoscopic mucosal resection (EMR) and ESD because an accurate histological assessment is provided, and the risk of local recurrence is reduced.^7^ ESD should be preferred over EMR because of the increased rates of curative resection and recurrence‐free survival.^8^ In this case, the length of the tumor is 3 cm, and the width is half a circumference. En bloc curative resection with EMR was thus considered difficult and we instead chose ESD for the resection of ESCC.

In this case, neither EVs nor ESCC has recurred as of 5 years after completing the EIS and ESD.

Hsu reported ESD after EVL without massive bleeding. The short interval between EVL and ESD may have had a positive effect.^9^ A case of gastric ESD after EIS has been reported that inflammation and fibrosis were observed around the EIS injection site.^10^ This might be because cyanoacrylate was injected, or pressure hemostasis with balloon was not available for gastric varices. We demonstrated a successful treatment of ESCC on EVs without EVL and no submucosal fibrosis other than inside the occluded vessels and verified it histologically. ESD after EIS represents one choice for the treatment of early ESCC on EVs. Further cases of this combination of pathologies need to be accumulated to clarify efficient endoscopic treatment of ESCC on EVs.

## CONFLICTS OF INTEREST

Masayuki Kitano received honoraria for lectures from Olympus Co., Ltd. None of the other authors have any conflicts of interest to declare.
